# Adenovectored RSV prefusion glycoprotein + soluble glycoprotein combination immunization establishes persistent opsonophagocytic antibody response through IgG3

**DOI:** 10.3389/fimmu.2025.1609779

**Published:** 2025-07-22

**Authors:** Xin Tong, Ross Blanc, Deniz Cizmeci, Hadar Malca, Jaewon Kang, Christy Comeaux, Benoit Callendret, Arangassery Rosemary Bastian, Mehak Zahoor Khan, Galit Alter, Daniel Lingwood, Ryan P. McNamara

**Affiliations:** ^1^ Ragon Institute of Mass General Brigham, MIT, and Harvard, Cambridge, MA, United States; ^2^ Department of Immunology and Infectious Diseases, Harvard T. H. Chan School of Public Health, Boston, MA, United States; ^3^ Janssen Vaccines & Prevention B.V., Leiden, Netherlands

**Keywords:** prefusion glycoprotein, RSV, vaccine, adenovirus vector, antibody

## Abstract

**Introduction:**

Respiratory syncytial virus (RSV) causes significant lower-respiratory-tract disease in high-risk groups. A phase IIb trial showed that a combination vaccine of Ad26-vectored prefusion RSV F (Ad26.preF) plus soluble prefusion F protein (SpreF) was 80 % protective, yet the longevity and functionality of these humoral responses are unknown.

**Methods:**

Sera from vaccinated adults were analyzed 12 months after the primary dose and 28 days after a homologous booster at 1 year. IgG subclasses, isotypes, Fc-γ receptor (FcγR) binding, and Fc-dependent effector functions including opsinophagocytosis against RSV-F subtypes A and B were quantified via Systems Serology.

**Results:**

A single dose generated durable immunity: IgG3-driven opsonophagocytic activity and broad FcγR engagement (via IgG1, IgG2, and IgG3) persisted through 12 months without appreciable decay. The booster did not increase antibody titres, FcγR binding, or functional activity, indicating that the primary response had already plateaued.

**Discussion:**

One dose of Ad26.preF + SpreF elicits a long-lasting, functionally potent humoral response that is not further enhanced by a booster at 12 months, suggesting limited benefit from early re-vaccination. Continued follow-up will clarify the duration of protection and inform booster timing in vulnerable populations.

## Introduction

Respiratory syncytial virus (RSV) infections represent the most common causes of severe respiratory disease in older and immunocompromised adults, children, and those with certain chronic cardiac or pulmonary comorbidities (1, 2). Annually, RSV is estimated to cause more than 60 million acute respiratory infections in adults and children worldwide (3). A recent prospective global study found that RSV causes a disease burden in infected adults similar to or worse than influenza, as measured by clinical symptom scores and medical resource utilization (4). Older adults are at a particularly high risk for severe RSV-mediated disease; in the United States alone, there are an estimated 177,000 hospitalizations and 14,000 deaths due to RSV in adults aged ≥65 years annually (5, 6). The high RSV-associated morbidity in older adults may result from waning or impaired immune responses with age, referred to as “immune senescence” (7). Given the high morbidity and significant disease burden of RSV in older adults, an effective prophylactic vaccine is needed to protect this population.

The RSV fusion glycoprotein (RSV F) represents a key target for vaccine design, as it is the primary antigen recognized by virus-neutralizing antibodies isolated from human sera (8, 9). The RSV F protein facilitates viral entry by driving the fusion of viral and host cell membranes, undergoing an irreversible conformational change from the accessible prefusion (preF) form to the more stable post-fusion (postF) form (10). While early efforts for vaccine development targeting the postF protein showed limited efficacy, likely attributed to the dominant antigenicity of preF in eliciting neutralizing antibodies, recent attempts to induce virus-neutralizing antibodies have been focused on the mutagenesis-stabilized form of preF protein (11). In contrast to postF-derived antigens, RSV preF-based vaccine candidates have demonstrated high immunogenicity and efficacy in multiple preclinical models (12, 13).

Natural infections with RSV do not prevent frequent reinfections, even within weeks of recovery despite exhibiting a persistently high level of neutralizing antibodies (14). Although these antibodies are associated with protection in both animal studies and human research, new evidence highlights the significance of additional immune correlates in the context of RSV infections and vaccination. These include the CD4+ and CD8+ T cell activities (15–17), along with Fc-effector functions such as antibody-dependent cellular phagocytosis (ADCP), antibody-dependent complement deposition (ADCD), and antibody-dependent natural killer cell activation (ADNKA). Specific antibody isotypes, such as serum and mucosal IgA, as well as subclasses like IgG1 and IgG3, have also been recognized as potential correlates of protection for RSV immunity (18, 19).

With this expanded understanding, an effective RSV vaccine candidate needs to generate not only neutralizing antibodies but also a diverse and lasting range of Fc-effector functional profiles. The RSV Ad26.preF represents a recombinant, replication-incompetent adenovirus type 26 (Ad26) vector encoding a conformation-stabilized RSV preF protein that demonstrated robust humoral and cellular responses in animal models (Ad26.preF). The safety and immunogenicity of Ad26.preF has been evaluated in several clinical trials. A single dose of Ad26.preF induced durable immunity in older adults (age ≥60 years) and demonstrated protection against RSV in a recent human challenge study of adults (ages 18–50 years) (20). When combined with recombinant stabilized RSV preF protein component (SpreF), this combination vaccine regimen demonstrated 80% efficacy in preventing RSV-related lower respiratory tract diseases in older adults (21). A previous study demonstrated that immune responses induced by a single regimen of the AD26.preF/SpreF combination vaccine waned over 12 months, and a booster appeared to be necessary to provide durable immune responses that would afford lasting protection against RSV (22). In the clinical study, a second dose of the same AD26.preF/SpreF combination vaccine was given on day 365 to evaluate the clinical impact of homologous boosting but was unable to explore the longitudinal profiles of antibody Fc-mediated functions associated with multiple vaccinations.

In this study, we systematically compare the vaccine-induced RSV-preF-specific antibody subclasses, isotypes, Fcγ receptor (FcγR) binding, and Fc-effector functions in participants receiving only one dose of AD26.preF/SpreF protein combination vaccine on day 1 and participants receiving the same combination regimen on both day 1 and month 12 in a phase I/IIa study. This analysis will clarify the functional roles of various immune components beyond B cells in providing sustained protection against RSV over an extended time while also contributing to immune persistence from repeated infections and vaccinations, which represents a unique feature of RSV’s virology.

## Methods and materials

### Study specimens

The serum samples were collected from a subgroup of the participants enrolled in a phase I/IIa clinical study (NCT03502707, cohort 3) that evaluates the safety and immunogenicity of AD26.preF/SpreF protein combination vaccine regimens in adults aged ≥60 years. The secondary use of samples in this study was approved by the Mass General Brigham Healthcare Institutional Review Board. Not human subjects research was determined by the MGH IRB. Identifiable information was not obtained nor used for this study. On day 1, the participants received the AD26.preF/SpreF protein vaccine (150 µg, high-dose, HD, *n* = 42) and placebo (*n* = 4). On day 365, a subset of participants received a second booster of AD26.preF/SpreF protein vaccine (150 µg, high-dose, HD, *n* = 28) and placebo (*n* = 18). Blood samples were collected pre-vaccination on days 1 and 29, 365, and 393 for evaluation of RSV-preF–specific antibody subclasses and isotypes, FcγR receptor binding, and Fc-effector functions. In this follow-up study, fluctuations in antibody titers and Fc-effector functions following multiple vaccinations were measured and evaluated as the geometric mean fold increase (GMFI) from the baseline levels (day 1) with the 95% confidence intervals (CIs). The median age of all participants was 68 years <(>60–81<)>, including the high-dose group (median: 66 years, 61–85) and the placebo group (median: 70 years, 66–82).

### Vaccines, antigens, and biotinylation

The RSV preF antigens biotinylated at the C-terminus used in this study were provided by Janssen Vaccines & Prevention BV. The vaccines used in the original clinical study have previously been described (20). Briefly, the replication-incompetent, recombinant adenoviral vectors based on Ad26 system were designed and engineered. The Ad26.preF vector encodes the full-length codon-optimized F gene from RSV A2 stabilized in its prefusion conformation using amino acid substitutions. The stabilized RSV preF protein (SpreF) was expressed in a stable Chinese hamster ovary (CHO) cell line. Subsequently, single-cell clones were isolated by semi-solid media cloning, expanded, and rank-ordered based on expression titer and product quality. For the selected lead clone, master and working cell banks were generated. Correct folding of the RSV preF antigen was determined by ELISA. The impurities and potential contaminants were controlled by Janssen Vaccines & Prevention BV during vaccine design and development (21). No adjuvant component was included in the formulation of this AD26.preF/SpreF protein combination vaccine regimen of both the single-dose and the booster vaccine regimen used in this study.

### Antibody isotype and Fc receptor binding

Antigen-specific antibody isotype and subclass titers and FcγR binding profiles were evaluated using a custom multiplex Luminex assay as previously described (37). Briefly, biotinylated RSV preF antigen was coupled to Luminex beads (Luminex Corp., Austin, TX, USA) with streptavidin (Jackson ImmunoResearch Inc, West Grove, PA, USA). The coupled beads were incubated with diluted serum samples, washed, and stained using diluted (1:100) phycoerythrin (PE)-conjugated secondary antibodies (Southern Biotech, Birmingham, AL, USA) for IgG1 (clone: Hp6001), IgG2 (clone: 31-7-4), IgG3 (clone: HP6050), IgG4 (clone: HP6025), IgM (clone: SA-DA4), IgA1 (clone: B3506B4), or IgA2 (clone: A9604D2). For FcγR binding, a biotinylated PE-streptavidin coupled recombinant human FcγR protein (Agilent Technologies, Santa Clara, CA, USA) was used as the secondary probe. After 1 h of incubation, the samples were washed, and relative antigen-specific antibody levels were quantified using an iQue^®^ analyzer (IntelliCyt, Albuquerque, NM, USA). All antibody levels and FcγR binding are reported as median fluorescence intensity (MFI).

### Antibody-dependent complement deposition

Antibody-dependent complement deposition (ADCD) assays were performed as described previously (23). Briefly, biotinylated RSV preF antigen was coupled to FluoSphere™ NeutrAvidin beads (Thermo Fisher) and incubated with 10 µL of diluted (1:250) serum samples or RSV preF IgG1 monoclonal antibody (Janssen) for 2 h at 37°C to form immune complexes. Nonspecific antibodies were removed by washing, and immune complexes were incubated with guinea pig complement (Cedarlane Laboratories, Burlington, Canada) in GVB++ buffer (Boston BioProducts, Inc., Milford, MA, USA) for 20 min at 37°C; the complement reaction was stopped by the addition of EDTA (15 mM in phosphate-buffered saline). Complement factor C3 deposited on beads was stained with anti-guinea pig C3-FITC antibody (MP Biomedicals, Irvine, CA, USA) and quantified using an iQue^®^ analyzer (IntelliCyt). ADCD was reported as MFI.

### Antibody-dependent neutrophil phagocytosis

Antibody-dependent neutrophil phagocytosis (ADNP) was evaluated using a phagocytosis score as previously described (23). Briefly, biotinylated RSV preF antigen was coupled to FluoSphere™ NeutrAvidin beads (Thermo Fisher) and incubated with 50 µL of diluted (1:1,000) serum samples for 2 h at 37°C to form immune complexes. Whole-blood samples were obtained from healthy donors and lysed with ammonium-chloride-potassium (ACK) buffer to isolate primary human neutrophils. The neutrophils were incubated with washed immune complexes for 1 h at 37°C, stained with diluted (1:100) Pacific Blue conjugated anti-CD66b antibody (BioLegend, San Diego, CA, USA; clone: G10F5), fixed with 4% paraformaldehyde solution, and analyzed using an iQue^®^ analyzer (IntelliCyt).

### Antibody-dependent cellular phagocytosis

Antibody-dependent cellular phagocytosis (ADCP) was evaluated using a phagocytosis assay with THP-1 cells as described previously (23). Briefly, biotinylated RSV preF antigen was coupled to FluoSphere™ NeutrAvidin beads (Thermo Fisher) and incubated with 50 µL of diluted (1:5,000) serum samples for 2 h at 37°C to form immune complexes. THP-1 monocytes were added to the beads and incubated for 16 h at 37°C. The samples were fixed with 4% paraformaldehyde solution and analyzed on an iQue^®^ analyzer (IntelliCyt). ADCP was reported as a phagocytosis score.

### Antibody-dependent natural killer cell activation

MaxiSorp™ ELISA plates (Thermo Fisher) were coated with RSV preF antigen for 2 h at room temperature and blocked with 5% bovine serum album (BSA; Sigma-Aldrich, St. Louis, MO, USA). Diluted (1:500) serum samples (100 µL) were added to the wells and incubated overnight at 4°C. Natural killer (NK) cells were isolated from buffy coats from healthy donors using the RosetteSep™ NK Cell Enrichment Kit (STEMCELL Technologies, Cambridge, MA, USA) and stimulated with recombinant human IL-15 (1 ng/mL; STEMCELL Technologies) at 37°C overnight. NK cells were added to the washed ELISA plate and incubated together with anti-human CD107a (BD Biosciences; clone: H4A3; Pe-Cy5; 1:40 dilution), brefeldin A (Sigma-Aldrich, St. Louis, MO, USA), and monensin (BD Biosciences) for 5 h at 37°C. The cells were surface-stained for CD56 (BD Biosciences; clone: B159; Pe-Cy7; 1:200 dilution), CD16 (BD Biosciences; clone: 3G8; APC-Cy7; 1:200 dilution), and CD3 (BD Biosciences; clone: UCHT1; Pacific Blue; 1:800 dilution). The cells were fixed and permeabilized using the FIX & PERM Cell Permeabilization Kit (Thermo Fisher) and stained for intracellular MIP-1β (BD Biosciences; clone: D21-1351; PE; 1:50 dilution) and IFN-γ (BD Biosciences; clone: B27; FITC; 1:17 dilution). NK cells were defined as CD3–/CD16+/CD56+, and frequencies of degranulated (CD107a+), MIP-1β+, and IFN-γ+ NK cells were quantified using an iQue^®^ analyzer (22).

### Statistical analysis

Data analysis was performed using GraphPad Prism (v9.2.0) and RStudio (v1.3 and R v4.0). Prior to analysis, all data were normalized using z-scoring. Statistical comparisons between groups were performed using Mann–Whitney *U*-tests followed by Benjamini–Hochberg (BH) or Bonferroni corrections to adjust for the number of comparisons made. Regression models were built using the R package “stats”. Correlations between immune response features at day 15 were performed using the Spearman method with BH-adjusted *P*-values. Univariate comparisons were done using Mann–Whitney *U*-test followed by Bonferroni correction. For univariate plots, statistical significance (*q*-value) was shown using *q < 0.05, **q < 0.01, and ***q < 0.001. Non-significant comparisons were left blank with the exception of the boosted comparisons, where n.s. = not statistically significant after Bonferroni correction (*q*-value > 0.05).

## Results

### Ad26.preF/RSV preF protein combination vaccine elicits a durable and functionally leveraged antibody response against RSV F

**Figure 1:**
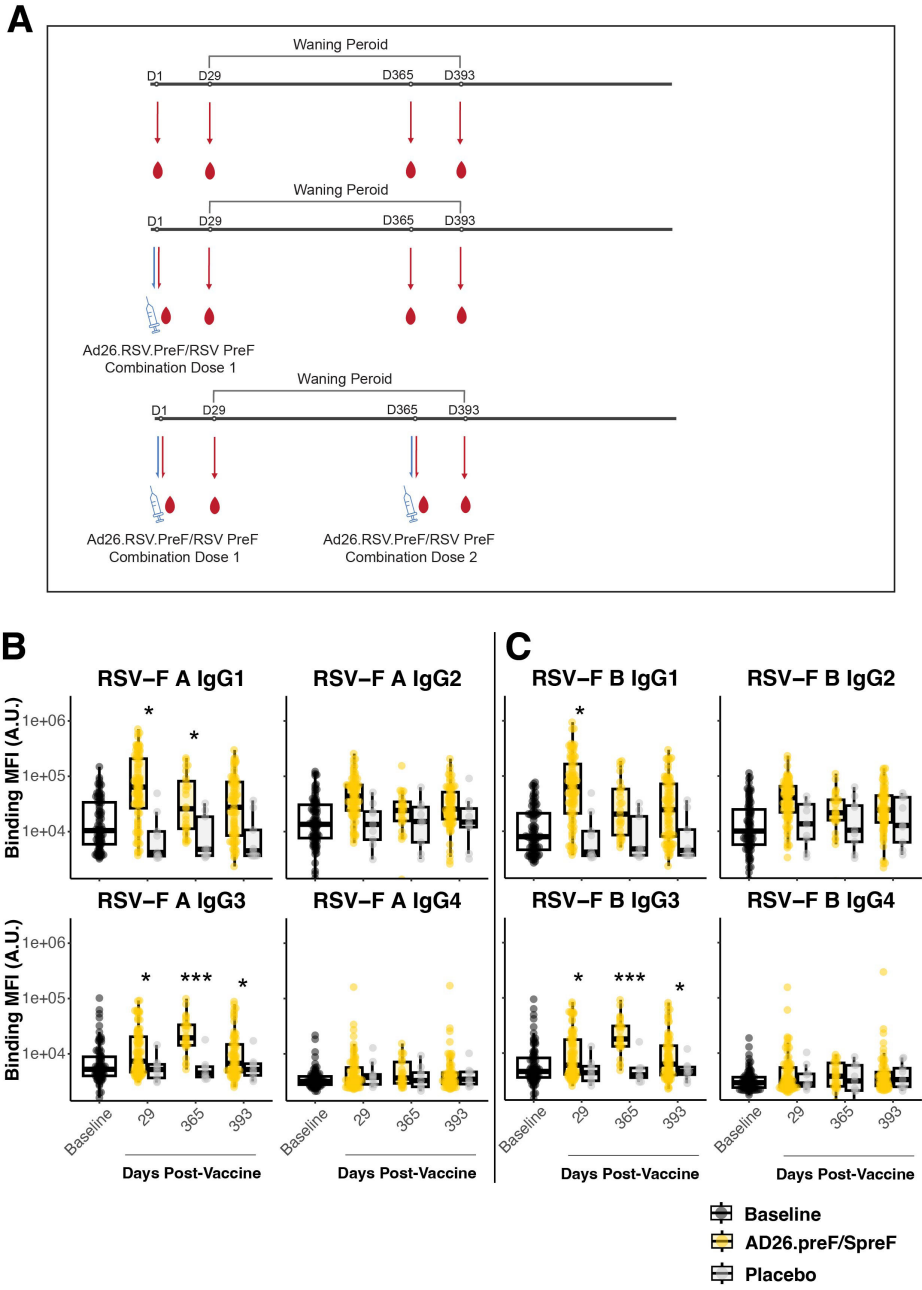
The AD26.preF/SpreF protein combination vaccine establishes a longitudinally preserved IgG3 response to RSV/A F and RSV/B F. **(A)** Treatment schematic. The participants had serum drawn immediately before vaccination with combinatorial vaccine AD26.preF/SpreF protein. The serum samples were subsequently collected on days 29, 365, and 393. A subset of participants who received an initial AD26.preF/SpreF protein was boosted with a second dose of the same combination vaccine on day 365. **(B)** Univariate comparisons of baseline (black), placebo-treated (gray), or AD26.preF/SpreF protein-vaccinated (gold) participants at the indicated timepoints for IgG1, IgG2, IgG3, and IgG4 against RSV F subtype A. **(C)** The same as **(B)** but for RSV F subtype B. Statistical comparisons were done using an initial Wilcox test followed by a Bonferroni correction for multiple comparisons. Comparisons were made for recipients of AD26.preF/SpreF protein against placebo at the same timepoint. Bonferroni-corrected *p*-values (*q*-values) were represented above the timepoints with *q < 0.05, **q < 0.01, and ***q < 0.001; comparisons whose *q*-value was ≥ 0.05 were left unlabeled. Data was plotted as a box and whisker showing median, interquartile ranges, and ranges. Individual data points were superimposed on each box and whisker plot. Values were plotted as binding mean fluorescence units (MFI) quantified as arbitrary units (A.U.) via multiplexed flow cytometry.

Specimens were taken from participants either placebo-treated or administered the adenovirus 26 vectored prefusion glycoprotein combined with the soluble prefusion glycoprotein (Ad26.preF/RSV preF protein) vaccine and followed for 1 year to evaluate peak and long-term humoral responses. Antibody responses to RSV subtype A and B fusion proteins (RSV-F A and RSV-F B) were characterized through systems serology at baseline (pre-treatment), day 29 (peak responses), day 365, and day 393 (**Supplementary Figure S1**; **Figure 1A**). IgG subclasses IgG1 and IgG3 showed a significant peak response post-Ad26.preF/RSV preF protein vaccination to RSV-F A over placebo, and IgG3 remained significantly induced up until day 393 (**Figure 1B**, compare yellow and gray groups). A similar trend was observed for RSV subtype B (**Figure 1C**). However, IgG1 binding levels to RSV-F B did not retain statistical significance at day 365. IgA showed a significant peak phase response that waned with time (**Supplementary Figure S2A**). *De novo* affinity maturation responses, as quantified through IgM, showed no significant differences at any timepoint for either treatment group for RSV-F A or RSV-F B protein (**Supplementary Figure S2B**). This would indicate that IgG and IgA responses were predominantly anamnestic-based (14).

**Figure 2:**
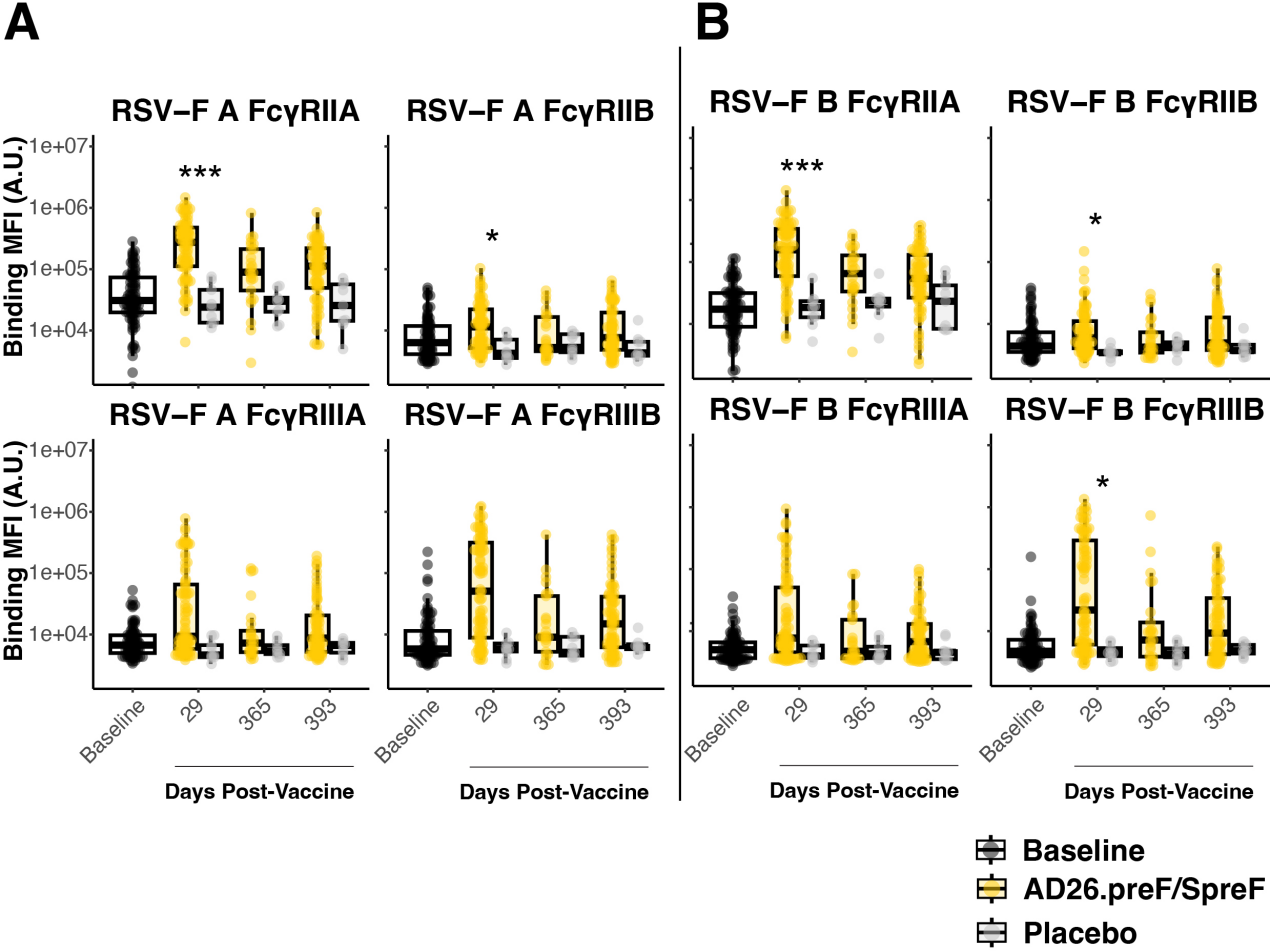
The AD26.preF/SpreF protein induces an acute FcγR-binding repertoire against both RSV/A F and RSV/B F. **(A)** Univariate comparisons of baseline (black), placebo-treated (gray), or AD26.preF/SpreF protein-vaccinated (gold) participants at the indicated timepoints for FcγRIIA, FcγRIIB, FcγRIIIA, and FcγRIIIB against RSV F subtype A. **(B)** Same as **(A)** but for RSV-F subtype B. Statistical comparisons were done using an initial Wilcox test followed by a Bonferroni correction for multiple comparisons. Comparisons were made for recipients of AD26.preF/SpreF protein against placebo at the same timepoint. Bonferroni-corrected *p*-values (*q*-values) were represented above the timepoints with *q < 0.05, **q < 0.01, and ***q < 0.001; comparisons whose *q*-value was ≥ 0.05 were left unlabeled. Data was plotted as a box and whisker showing median, interquartile ranges, and ranges. Individual data points were superimposed on each box and whisker plot. Values were plotted as binding mean fluorescence units (MFI) quantified as arbitrary units (A.U.) via multiplexed flow cytometry.

Antibody effector functions depend on an antibody’s Fc region binding to Fc-receptors. IgG subclasses bind to Fc-gamma receptors (FcγR), while IgA subclasses bind to Fc alpha receptors (FcαR) (23). FcγR-binding antibodies to RSV-F showed strong initial responses in Ad26.preF/RSV preF protein vaccinees. After adjustment for multiple comparisons, only day 29 for RSV A and RSV B showed elevated responses relative to placebo-treated participants (**Figures 2A, B**). Interestingly, the exception was FcγRIIIA, which is an activating receptor on the surface of natural killer cells. Neither RSV A nor RSV B showed any statistically significant induction even at peak immunogenicity after Ad26.preF/RSV preF protein vaccination. However, even though it did not achieve statistical significance, there was a clear trend for the recipients of Ad26.preF/RSV preF protein for a sustained FcγR-binding antibody profile. FcαR-binding antibodies showed only an acute-phase response post-vaccination that did not achieve statistical significance after multiple-comparisons adjustment (**Supplementary Figure S3**).

### Ad26.preF/RSV preF protein provides long-term opsinophagocytic effector function

**Figure 3:**
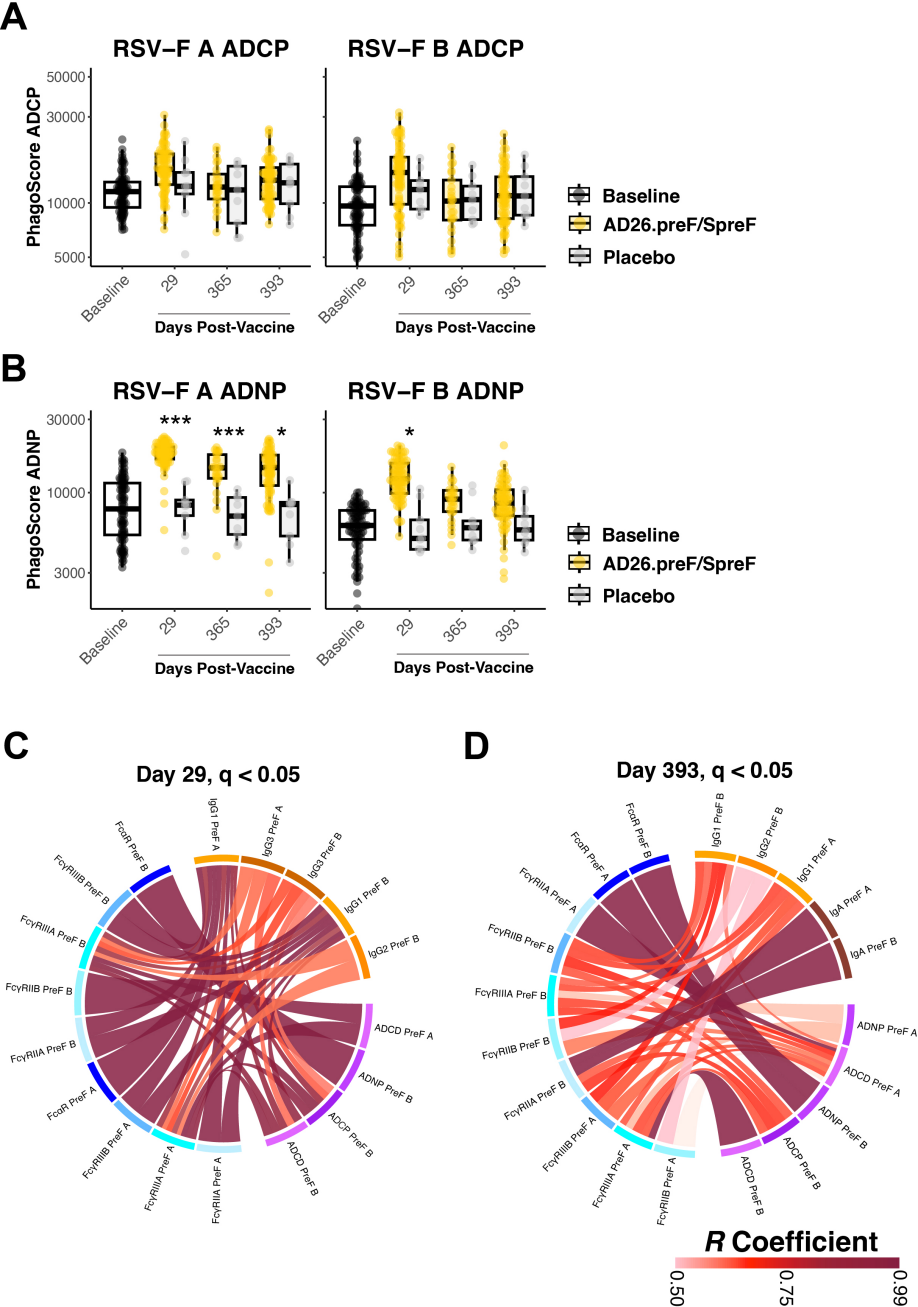
The AD26.preF/SpreF protein induces a significant opsinophagocytic response and coordinated humoral profiles for over 1 year. **(A)** Antibody-dependent cellular phagocytosis (ADCP) was quantified at baseline (black), placebo-treated (gray), or AD26.preF/SpreF protein-vaccinated (gold) participants at the indicated timepoints for RSV/A F (left) and RSV/B F (right). **(B)** Antibody-dependent neutrophil phagocytosis (ADNP) was quantified at baseline (black), placebo-treated (gray), or AD26.preF/SpreF protein-vaccinated (gold) participants at the indicated timepoints for RSV/A F (left) and RSV/B F (right). **(C)** Correlation ribbon wheel to show statistically significant Spearman’s correlations (*q*-value < 0.05) between humoral features at day 29 post-vaccination with AD26.preF/SpreF protein. **(D)** Same as **(C)** but for correlations at day 393, the last draw date for recipients of the AD26.preF/SpreF protein (single dose); the legend for Spearman’s correlation coefficient value is shown in the bottom right. Comparisons were made for recipients of AD26.preF/SpreF protein against placebo at the same timepoint. Bonferroni-corrected *p*-values (*q*-values) were represented above the timepoints with *q < 0.05, **q < 0.01, and ***q < 0.001; comparisons whose *q*-value was ≥ 0.05 were left unlabeled. Data was plotted as a box and whisker showing median, interquartile ranges, and ranges. Individual data points were superimposed on each box and whisker plot. Values were plotted as the defined phagoscore, which is a measurement of opsinophagocytosis of fluorescent beads.

Antibody effector functions such as antibody-dependent cellular phagocytosis by monocytes (ADCP) and antibody-dependent neutrophil phagocytosis (ADNP) are linked with FcαR- and FcγR-binding antibodies (24, 25). We thus profiled how these effector functions longitudinally responded to Ad26.preF/RSV preF protein vaccination. ADCP for RSV-F A and RSV-F B proteins showed an acute phase response in Ad26.preF/RSV preF protein**-**vaccinated participants that waned to baseline and placebo-treated values over 1 year (**Figure 3A**). In contrast, ADNP to RSV A was sustained for 393 days over baseline and placebo in the Ad26.preF/RSV preF protein-vaccinated participants; ADNP to RSV B was strongly induced at day 29 but waned with time (**Figure 3B**). This is in agreement with elevated, activating FcγR-binding antibodies within this subgroup. Antibody-dependent complement deposition (ADCD) response was significantly more pronounced for RSV B than for RSV A and was sustained above the baseline level and placebo for 393 days post-Ad26.preF/RSV preF vaccination (**Supplementary Figure S4**).

We next asked how the humoral features were linked following Ad26.preF/RSV preF protein vaccination. We generated correlation chord diagrams showing correlated features whose Spearman’s correlation coefficient was >0.5 and was statistically significant after Bonferroni correction (*q* value < 0.05). We evaluated how the humoral profile was linked at day 29 post-vaccination, which would be at or nearest to peak immunogenicity, and at day 393, which would represent a settled response. Binding antibodies (orange blocks) were tightly correlated with both FcR-binding antibodies (blue blocks) and effector functions (purple blocks) 29 days after Ad26.preF/RSV preF protein vaccination (**Figure 3C**). Only IgG subclasses were selected on day 29. No IgM-based correlations were observed to exceed the pre-selected cutoffs, again supporting that Ad26.preF/RSV preF protein was promoting an anamnestic response and not a *de novo* antibody response. At day 393, multiple significant correlations persisted; however, IgA was also present within our correlation chords, linking with FcγRIIA, which was also linked to ADCP and ADCD (**Figure 3D**). Within the correlation chord diagram, both RSV-F A and B antigens were selected, demonstrating that the Ad26.preF/RSV preF protein combination elicits cross-reactive and polyfunctional antibodies that are detectable for more than 1 year.

### Humoral networks established by Ad26.preF/RSV preF protein vaccination are highly persistent

At day 365 post-vaccination with Ad26.preF/RSV preF protein, a subset of participants was boosted with a second dose of the combination Ad26.preF/RSV preF protein vaccine. Therefore, day 393 would represent a peak response of the boosted individuals. We thus analyzed how the humoral profiles were shaped after the boost and how they compared with the non-boosted participants.

**Figure 4:**
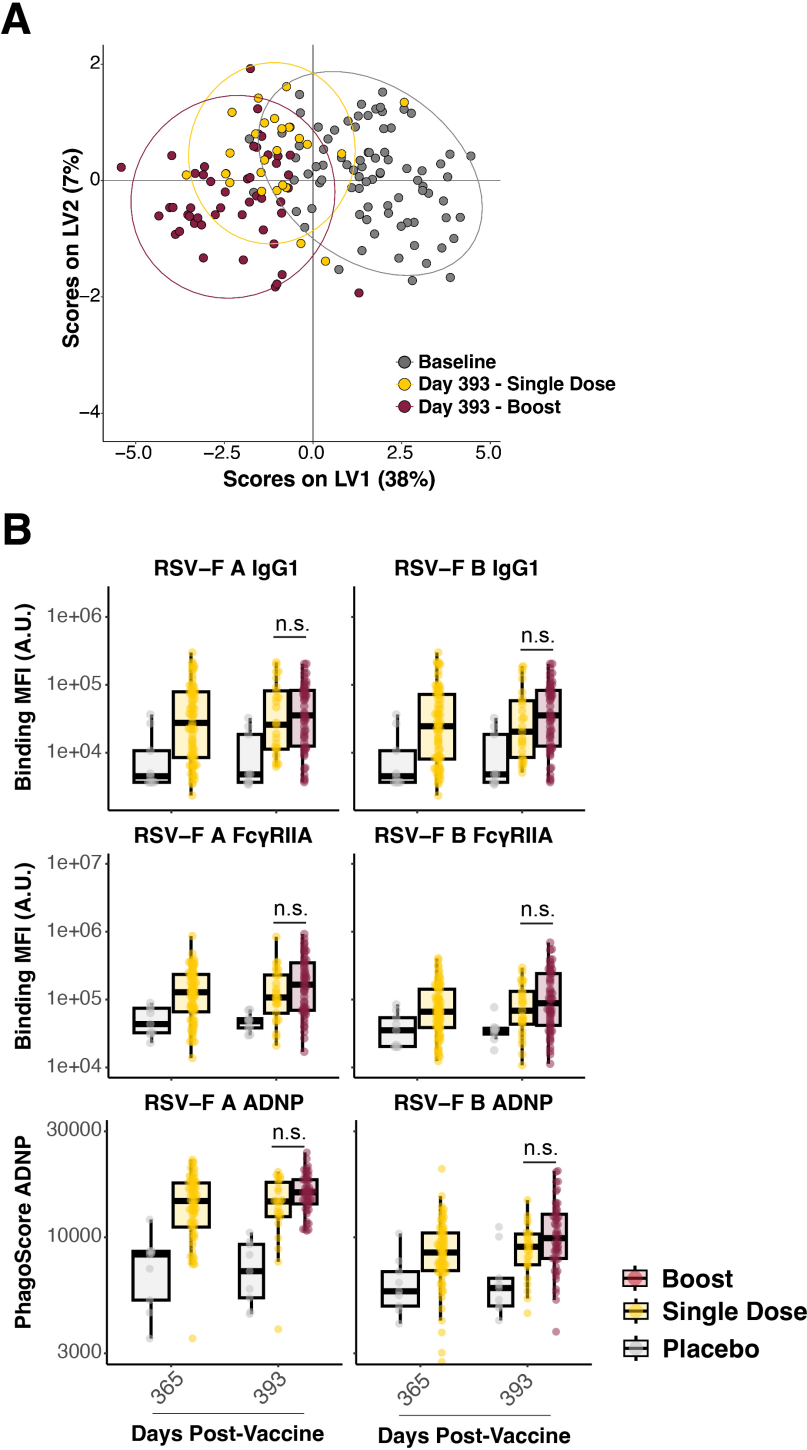
Boosting with AD26.preF/SpreF protein demonstrated negligible effects on AD26.preF/SpreF protein-primed recipients. **(A)** Multivariate clustering using partial least squares discriminate analysis (PLSDA) of individuals at baseline (black), day 393 single dose (gold), and day 393 boosted (red). Baseline humoral profiles were used instead of placebo for multivariate clustering to improve model separation. **(B)** (Rows 1 and 2) Univariate comparisons of placebo-treated (gray), single-dose AD26.preF/SpreF protein-vaccinated (gold), or boosted (red) participants at the indicated timepoints for IgG1 and FcγRIIA against RSV/A F (left) and RSV/B F (right). (Row 3) ADNP for placebo-treated (gray), single-dose AD26.preF/SpreF protein-vaccinated (gold), or boosted (red) participants at the indicated timepoints for RSV/A F (left) and RSV/B F (right). Bonferroni-corrected *p*-values (*q*-values) were represented above the timepoints with *q < 0.05, **q < 0.01, and ***q < 0.001; comparisons whose q-value was ≥ 0.05 were labeled as n.s. for not significant. Data was plotted as a box and whisker showing median, interquartile ranges, and ranges. Individual data points were superimposed on each box and whisker plot.

Multivariate signatures showed a separation between the baseline (gray) and the single-dose Ad26.preF/RSV preF protein at day 393 (yellow); however, signatures between the single-dose and the boosted group (red) showed a considerable overlap (**Figure 4A**). At the univariate level, binding IgG1 and FcγRIIA antibodies to both RSV-F A and B were comparable between the single-dose and boosted group, with both remaining elevated over placebo. These were the features that exhibited the greatest sustained separation from baseline, and we thus thought that any differences in the boost would be highlighted among these. However, no statistically significant increase in antibody levels was observed (**Figure 4B**, rows 1 and 2). Thus, no observable anamnestic or expanded response could be identified through boosting with Ad26.preF/RSV preF protein.

A similar resistance to the recall/memory and the expansional functional output as quantified by ADNP was observed in the boosted group (**Figure 4B**, row 3). This aligns with the binding, FcγR-binding, and multivariate clustering, showing high degrees of overlap between the single dose Ad26.preF/RSV preF protein and the boosted group. Taken together, the Ad26.preF/RSV preF protein vaccine elicited a humoral response that remained largely coordinated for up to 393 days post-vaccination. This antibody Fc-mediated functional coordination remained even though the antibody levels waned over time.

## Discussion

Despite several attempts at developing a protective vaccine against RSV infections (26), the currently available treatments and vaccines are still limited (27, 28). While neutralizing antibodies are often used as indicators of immunity in the context of many approved vaccines, simply measuring antibody levels has not provided sufficient insight to guide vaccine development for several pathogens, including RSV (28–32). Recent research underscores the importance of antibody functionality over quantity as a key factor in infectious immunity, though the specific immune markers for protection against RSV are not yet well understood (33). Although neutralizing antibodies have been linked to immunity in some RSV studies, this connection is not consistently strong across all epidemiological data. Similarly, while ADCC activity has shown protective effects in animal models, it has not yet been proven as a reliable measure of immunity in humans (34). To better understand this, we conducted a study using a unique RSV vaccine regimen, AD26.preF/SpreF, which had shown promising antibody responses in previous trials (20, 21). To our knowledge, these results represent the first study in which antibody Fc-mediated functionality induced by homologous vaccine boosting was evaluated in a multivariate manner regarding the AD26.preF/SpreF combination vaccine. Our goal was to thoroughly examine both humoral and Fc-mediated innate immune responses induced by the two-dose AD26.preF/SpreF regimen compared to the single-dose and placebo controls.

In this study, we compared the antibody profiles induced by three vaccination approaches: two doses of AD26.preF/SpreF combination vaccine, one dose of the same vaccine, and a placebo. Consistent with earlier research, a single dose of AD26.preF/SpreF led to a notable increase in RSV-preF-specific IgG1 and IgG3 levels, enhanced Fc receptor interactions, and improved Fc-mediated functions (27). These peak immune responses persisted for up to 365 days. Notably, these antibodies showed significant engagement with Fcγ receptors, leading to sustained opsonophagocytic activity, particularly antibody-dependent neutrophil phagocytosis (ADNP) and complement deposition (ADCD) for more than 1 year. These functions are critical for effectively clearing RSV infections, highlighting the importance of non-neutralizing antibody functions in vaccine efficacy (35).

Surprisingly, a booster dose of AD26.preF/SpreF administered 1 year following the initial vaccination did not significantly enhance the overall immune response profiles. Multivariate analyses revealed that the immune profile of participants receiving the booster largely overlapped with those who received only the single dose. This suggests that the initial vaccination established the initial immune system in a way that limited further expansion of the antibody response upon boosting. While the lack of an anamnestic response challenges conventional booster strategies, it underscores the potential of a single-dose regimen for providing long-term protection. These findings present significant implications for future RSV vaccine strategies. Future studies should investigate the mechanisms of the observed immune persistence and explore the alternative dosing schedules, including earlier or later homologous boosts, or novel formulations, to enhance long-term protection.

Currently, several RSV vaccines have been approved for use in older adults, including *Arexvy* (GSK), *Abrysvo* (Pfizer), and *mRNA-1345* (Moderna, mRESVIA). *Arexvy* and *Abrysvo* are both approved for individuals aged 60 years and older, with *Abrysvo* also approved for maternal immunization during pregnancy to confer passive protection to infants. *mRESVIA* is an mRNA-based vaccine recently approved in the United States for adults aged 60 years and older (23). These licensed vaccines demonstrated robust neutralizing antibody responses targeting the RSV prefusion F protein. In this study, we characterize immune profiles generated by the Ad.26.preF/SpreF at the systems level. Through this approach, we identified that this vaccination platform elicits a durable and polyfunctional response characterized by sustained IgG3 production, strong Fcy receptor engagement, and long-lasting opsinophagocytic functions. This effector-driven profile may offer complementary protection to other vaccine platforms more focused on neutralization, particularly in older adults where immune senescence can limit the effectiveness of neutralizing responses alone. Future studies are needed to more comprehensively compare the immune features across the different RSV vaccine platforms and assess their unique correlates to clinical protection in diverse populations.

While this study provides valuable insights into the durability and functional quality of the AD26.preF/SpreF-induced antibody responses, exploring additional immune compartments could further improve our understanding of its protective mechanisms. Specifically, analysis of cellular immune responses, such as the roles of CD4+ and CD8+ T cells, could illuminate how T cells help influence antibody generation, isotype switching, and immune memory formation. Similarly, evaluating memory B cell populations may offer crucial insights into the long-term recall potential and specificity of antibody responses induced by RSV vaccination. Although antibody titers and Fc-mediated effector functions serve as important indicators of immune persistence, defining robust correlates of protection against RSV remains an important challenge. Future studies incorporating comprehensive analyses of T cell activity, memory B cell profiling, antigen-specific single-cell sequencing, and integrated systems serology approaches will likely yield valuable data to refine immunological benchmarks for RSV vaccine efficacy and guide licensure decisions.

In conclusion, these results present significant implications for future vaccine strategies against RSV, particularly in vulnerable populations such as older adults, infants, and the immunosuppressed, where immune senescence often complicates effective immunization against repeated infections by RSV (5). The relatively durable opsonophagocytic response induced by this combination vaccine over 1 year, even in the absence of globally significant booster effects, could inform the design of more effective RSV vaccines in terms of overcoming immune persistence (36). Future studies should focus on identifying the mechanisms behind immune persistence and exploring whether alternative dosing schedules or formulations could enhance long-term protection.

## Data Availability

The datasets presented in this study can be found in online repositories. The names of the repository/repositories and accession number(s) can be found below: https://github.com/HSPHSystemsSerology/XT20250409.
