# Supplementary material for: Adenovectored RSV prefusion glycoprotein + soluble glycoprotein combination immunization establishes persistent opsonophagocytic antibody response through IgG3
Source: Front Immunol. 2025 Jul 22;16:1609779. doi: 10.3389/fimmu.2025.1609779 (PMC12322566; doi:10.3389/fimmu.2025.1609779)
Supplement: Supplementary file 1 [file DataSheet1.pdf]

Supplementary Information

Supplementary Information contains Supplementary Figures 1-4 and their corresponding Supplementary Figure Captions.

Supplementary Figure 1

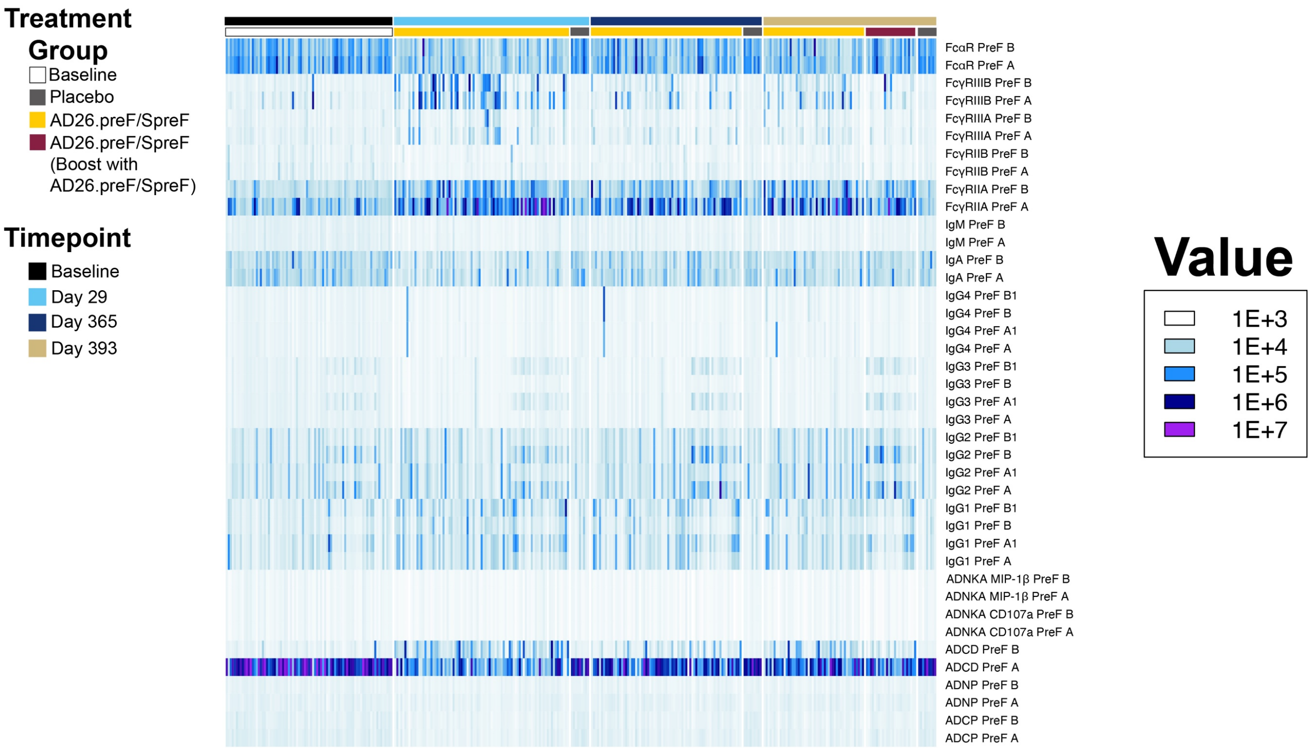

Supplementary Figure 1. Overall heatmap for systems serology analysis. Antibody binding and effector functions are shown in heatmap representation. Treatment group and timepoint coloring scheme is shown on the left. Relative values for the individual heatmap blocks are shown on the right.

Supplementary Figure 2

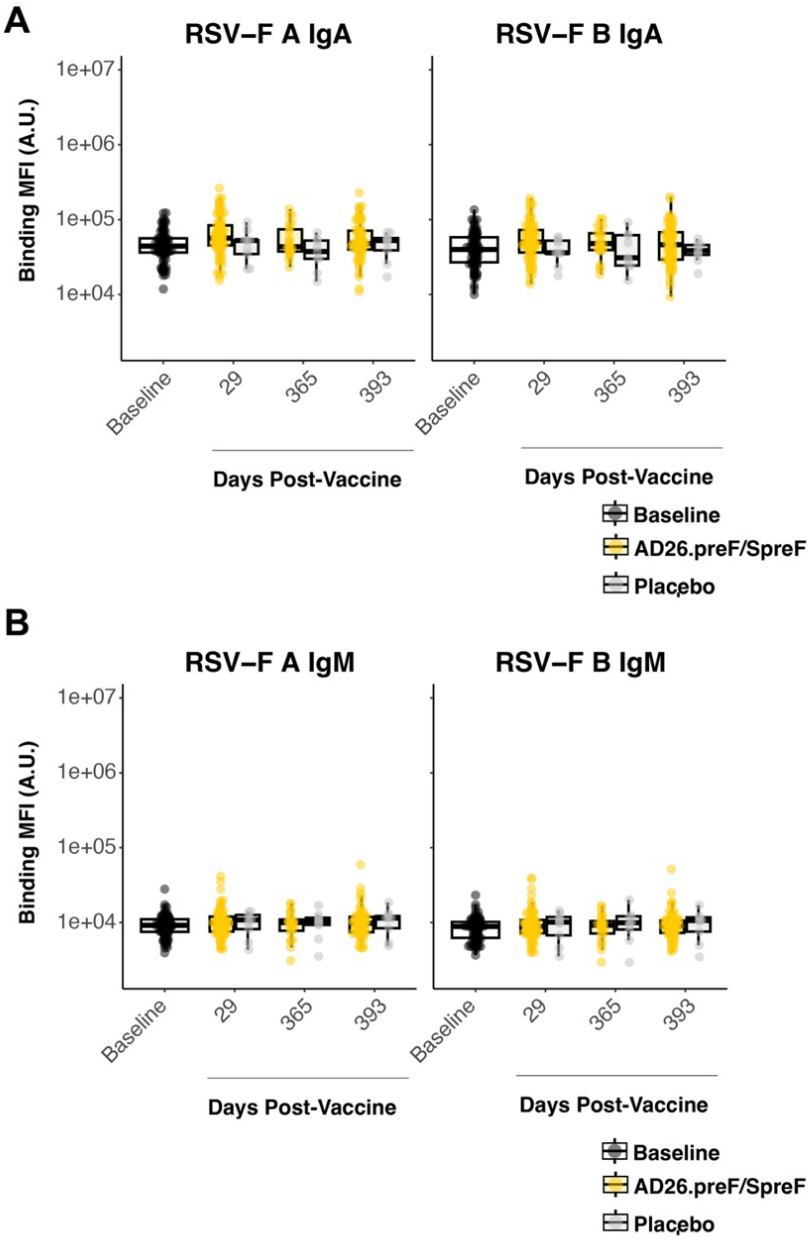

Supplementary Figure 2. IgA and IgM responses to AD26.preF/SpreF are muted. (A) Univariate comparisons of baseline (black), placebo-treated (gray), or Ad26.preF/SpreF protein-vaccinated (gold) participants at the indicated timepoints for IgA for RSV-F A (left) and RSV-F B. (B) Same as A, but for IgM. No statistical significance was observed.

Supplementary Figure 3

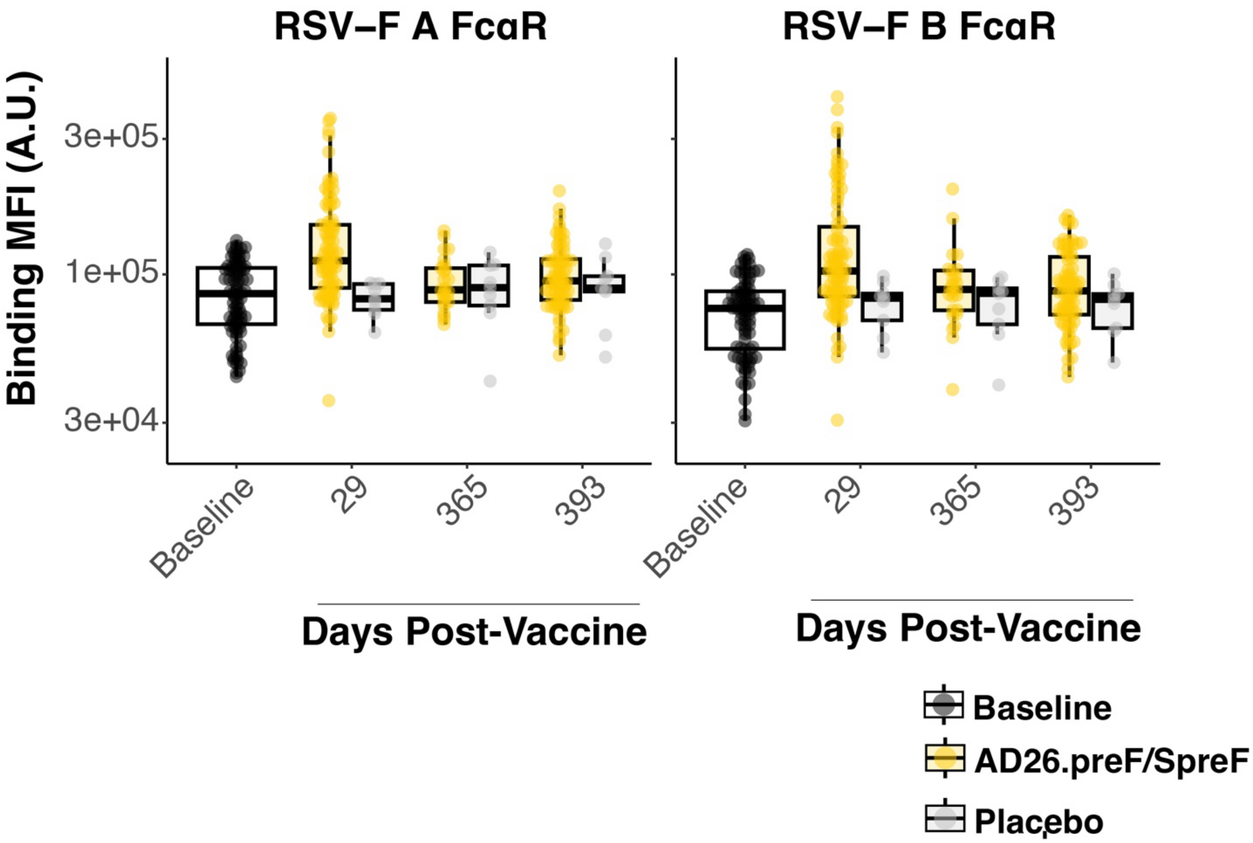

Supplementary Figure 3. FcαR-binding antibodies are not induced by AD26.preF/SpreF. Univariate comparisons of baseline (black), placebo-treated (gray), or Ad26.preF/SpreF protein-vaccinated (gold) participants at the indicated timepoints for FcαR-binding antibodies for RSV-F A (left) and RSV-F B. No statistical significance was observed.

Supplementary Figure 4

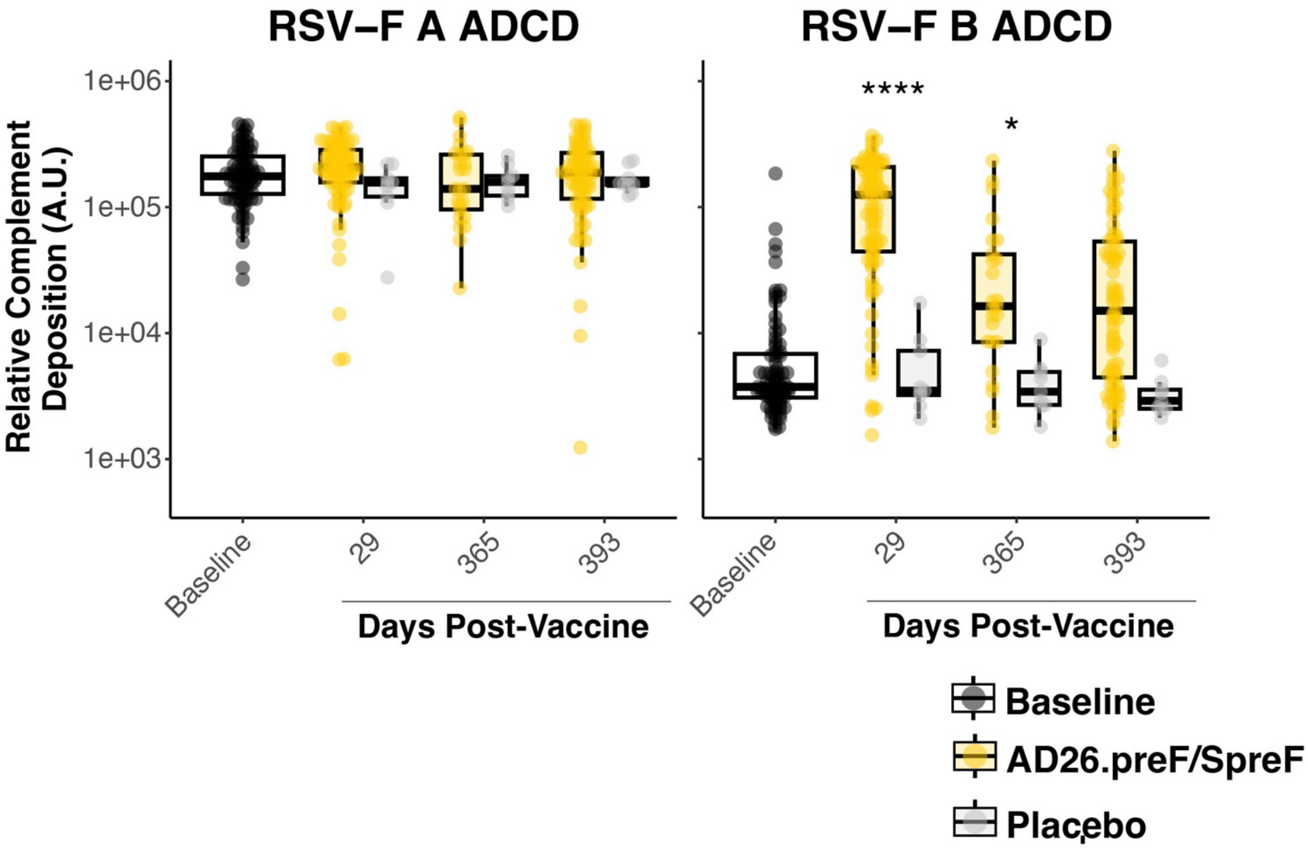

Supplementary Figure 4. Antibody-dependent complement deposition (ADCD) is induced only for RSV-F B. (A) Univariate comparisons of baseline (black), placebo-treated (gray), or Ad26.preF/SpreF protein-vaccinated (gold) participants at the indicated timepoints for ADCD for RSV-F A (left) and RSV-F B. No statistical significance was observed.
